# Effects of Cod-liver Oil on the Blood

**Published:** 1854-09

**Authors:** Theophilus Thompson


*Effects of Cod-liver Oil on the blood.*—A paper was read by the Royal Society:—"On the changes produced in the Blood at the Administration of Cod-liver Oil and Cocoa-nut Oil." By Theophilus Thompson, M. D., F. R. S.—The author has found, that during the administration of cod-liver oil to phthisical patients their blood grew richer in red corpuscles, and he refers to a previous observation of Dr. Franz Simon to the same effect. The use of almond-oil and of olive-oil was not followed by any remedial effect; but cocoa-nut oil results were obtained almost as decided as from the oil of the liver of the cod, and the author believes it may turn out to be a useful substitute. The oil employed was a pure cocoa oliene, obtained by pressure from crude cocoa-nut oil, as expressed in Ceylon and the Malabar coast from the Copperah or dried cocoa-nut kernel, and refined by being treated with an alkali, and then repeatedly washed with distilled water. It burns with a faint blue flame, showing a comparatively small proportion of carbon, and is undrying. The analysis of the blood was conducted by Mr. Dugald Campbell. The whole quantity abstracted having been weighed, the coagulum was drained on bibulous paper for four or five hours, weighed, and divided into two portions. One portion was weighed and then dried in a water oven to determine the water. The other was macerated in cold water until it became colorless, then moderately dried, and digested with ether and alcohol, to remove fat; and, finally, dried completely, and weighed as fibrin. From the respective weights of the fibrin, and the dry clot, that of the corpuscles was calculated. The following were the results observed in seven different individuals affected with phthisic in different stages of advancement:—

| | | Red corpuscles. | Fibrin |
|---|---|---|---|
| First stage, before the use of cod-liver oil, . | Female, | 129.26 | 4.52 |
| | Male, | 116.53 | 13.57 |
| First stage, after the use of cod-liver oil, . | Female, | 136.47 | 5.00 |
| | Male, | 141.53 | 4.70 |
| Third stage, after the use of cod-liver oil . | Male, | 138.74 | 2.23 |
| Third stage, after the use of cocoa-nut oil, . | Male, | 139.95 | 2.31 |
| | Male, | 144.94 | 4.61 |

